# Clustering of phosphorylation site recognition motifs can be exploited to predict the targets of cyclin-dependent kinase

**DOI:** 10.1186/gb-2007-8-2-r23

**Published:** 2007-02-22

**Authors:** Alan M Moses, Jean-Karim Hériché, Richard Durbin

**Affiliations:** 1 Wellcome Trust Sanger Institute, Wellcome Trust Genome Campus, Hinxton, Cambridge, CB10 1HH, UK

## Abstract

Protein kinases are critical to cellular signalling and post-translational gene regulation, but their biological substrates are difficult to identify. We show that cyclin-dependent kinase (CDK) consensus motifs are frequently clustered in CDK substrate proteins. Based on this, we introduce a new computational strategy to predict the targets of CDKs and use it to identify new biologically interesting candidates. Our data suggest that regulatory modules may exist in protein sequence as clusters of short sequence motifs.

## Abstract 2

A novel computational strategy is used to predict cyclin-dependent targets by exploiting their propensity for occurring in clusters on substrate proteins.

## Background

Protein kinases are ubiquitous components of cellular signalling networks [1]. A relatively well understood example is the network that controls progression of the cell cycle, where cyclin-dependent kinases (CDKs) couple with various cyclins over the cell cycle to regulate critical processes [2-4]. Despite their biological and medical importance, relatively few direct, *in vivo *targets of these kinases have been identified conclusively, because experimental techniques are difficult and time consuming [1,5]. With the availability of databases of protein sequences, computational methods provide an alternative approach [6,7].

Kinase substrates often have short, degenerate sequence motifs surrounding the phosphorylated residue [8]. Putative target residues can be predicted by searching for matches to the consensus for a particular kinase. For example, CDK substrates often contain S/T-P-X-R/K where X represents any amino acid, and S/T represents the phosphorylated serine or threonine [9,10]. Because of the low specificity of the CDK consensus, however, databases of protein sequences are expected to contain large numbers of matches by chance. Therefore, many of the matches in protein sequences are likely to be false-positive predictions. Consistent with this, when 553 *Saccharomyces cerevisiae *proteins with at least one match to the CDK consensus were tested in a high-throughput kinase assay, only 32% (178) were found to be substrates [11]. Furthermore, in some cases characterized CDK substrates are phosphorylated at residues matching only a minimal consensus S/T-P [12]; considering these weak matches would probably lead to even larger numbers of false positives.

Characterized CDK targets may be phosphorylated at multiple residues (for instance, see the report by Lees and coworkers [13]). Recent studies of several CDK target proteins in *S. cerevisiae *have shown that these multiple phosphorylations can regulate stability [12], protein interaction [14,15], or localization [16]. Motivated by these observations, we propose an alternative computational strategy to identify substrates of CDKs; instead of attempting to predict individual phosphorylation sites, we search for proteins that contain high densities of strong and weak consensus matches that are closely spaced in the primary amino acid sequence. (We refer to this close spacing as 'clustering', and this should not be confused with clustering of multivariate data.)

Taking advantage of the results of a high-throughput study [11], we show statistically that CDK1 targets in *S. cerevisiae *contain multiple closely spaced consensus matches and we develop computational methods to identify such proteins. We also find that these clusters tend to occur in disordered or unfolded regions near the termini of the protein. We show that it is possible to predict proteins that are likely to be targets of CDKs in *S. cerevisiae *by searching for proteins that contain clustered matches to the CDK consensus. We also show that human CDK targets are enriched for proteins that contain clustered consensus matches and, by searching human cell cycle genes, we predict several putative CDK targets, including the human orthologs of *Schizosaccharomyces pombe *CDC5 (CDC5L) and *S. cerevisiae *Cdc20p (CDC20). Finally, we examine co-clustering of the CDK consensus motifs with the 'cy' or RXL motif [17], which is known to be important in determining which CDK-cyclin complex will phosphorylate a given substrate.

## Results

### Targets of Cdk1p in *S. cerevisiae *contain clusters of matches to the CDK consensus

**Figure 1:**
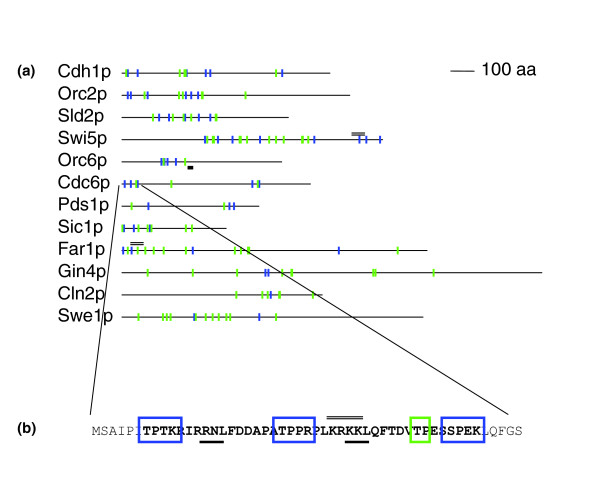
Clustering of consensus motifs in *S. cerevisiae *CDK targets. **(a) **Schematics of characterized *S. cerevisiae *CDK targets. Blue and green symbols indicate matches to the strong and weak CDK consensus, respectively. The thick black bar below indicates the characterized cy motif in Orc6. The double lines above indicate characterized nuclear localization signals. **(b) **Sequence of the amino-terminus of Cdc6. Blue and green boxes indicate matches to the strong and weak CDK consensus, respectively. Bold letters indicate the region with the maximal scoring cluster according to S_*BN*_. We suggest that this region may be regarded as a regulatory module (see text for details). Thick bars below the sequence indicate matches to the 'cy' motif and thin double lines above the sequence indicate characterized nuclear localization signals. aa, amino acid; CDK, cyclin-dependent kinase.

**Figure 2:**
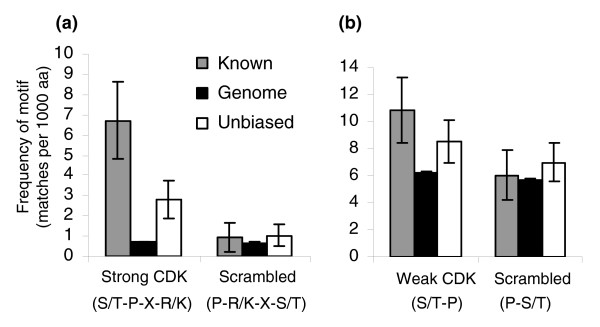
Enrichment of matches to the CDK consensus in CDK substrates. **(a) **The protein sequences of well characterized ('known') CDK targets (gray bars) are highly enriched for matches to the CDK strong consensus relative to the genome (black bars) but not for a scrambled version of the consensus. Similar results hold for the 'unbiased positives' from a high-throughput study (unfilled bars). **(b) **'Known' and 'unbiased positives' are also somewhat enriched for the weak consensus but not for a scrambled version of it. See text for details. Frequencies are number of matches per 1000 amino acid (aa) residues. Error bars represent plus or minus two times the standard error. CDK, cyclin dependent kinase.

CDK substrates in *S. cererevisiae *are often phosphorylated at multiple serine or threonine residues, some of which match the full (henceforth 'strong') consensus S/T-P-X-R/K, whereas others match a minimal (henceforth 'weak') consensus S/T-P. For example, the amino-terminal region of Cdc6p (Figure 1b) is a direct target of Cdk1p (also known as Cdc28p) [14], and contains three strong and one weak CDK consensus. In order to test whether these observations could be used to predict new substrates, we first compared the number of matches of each motif per residue in a set of 12 Cdk1p targets known from low-throughput biochemical and genetic experiments (compiled by Ubersax and coworkers [11]; henceforth referred to as 'known' targets; see Table 1 and Figure 1a) with the number in the genome. We find a highly significant, more than ninefold enrichment of the strong consensus (Figure 2a, left side) but not for a scrambled version (P-R/K-X-S/T) of the consensus (Figure 2a, right side), indicating that the enrichment is not due to simple compositional effects. For the weak consensus (after masking the strong consensus), we also find enrichment over the genome and not for a scrambled consensus (after masking the weak and strong consensus), but it is less striking (less than twofold; Figure 2b).

Because we were concerned that the discovery of the known targets may have been biased by the observation that they contained many matches to the strong consensus, we also computed these frequencies for the 18 proteins out of a set of 198 randomly chosen genes from *S. cerevisiae *identified as Cdk1p targets in a high-throughput assay [11] (henceforth referred to as 'unbiased positives'; see Table 1). We found similar results in this unbiased positive set, although the enrichment of strong matches was just under fourfold in this case and the enrichment of weak matches was less than 1.5-fold (Figure 2). That the fold enrichment is somewhat less in this set is consistent with some of the enrichment in the known set being due to bias in their discovery, but also with some false-positive findings being picked up in the kinase assay. Nevertheless, this rules out the possibility that the enrichment of matches in *bona fide *CDK substrates is only the result of a bias.

**Figure 3:**
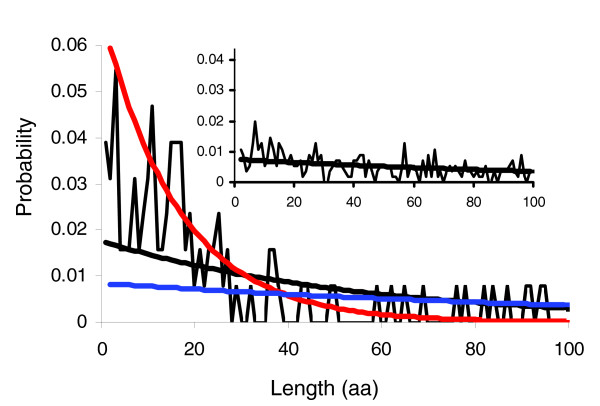
Modeling the distribution of spacing distances between matches to the CDK consensus. Fit of one (black trace) or two multivariate geometric components (blue and red traces) to the observed spacings (thin black trace) in the 'known' targets. The 'known' targets exhibit an excess of short spacings over what would be expected under the single geometric. The inset shows the geometric fit (black trace) to the spacings observed (thin black trace) in the 'unbiased negatives' and shows much better agreement. See text for details. CDK, cyclin-dependent kinase.

Examination of phosphorylated residues in CDK target proteins reveals that they are often found 'clustered' in one region of the primary amino acid sequence (Figure 1). We sought to test whether this apparent clustering was due simply to a uniform overall enrichment of consensus matches in these proteins, or whether it was a preference for the consensus matches to occur near each other. We modeled the number of residues until a strong or weak match was identified using a bivariate geometric distribution (see Materials and methods, below). We then performed a likelihood ratio test (LRT) between the hypothesis (*H*_*1*_) that the spacings were drawn from a mixture of a high-density 'cluster' component and a low-density 'background' component, and the hypothesis (*H*_*0*_) that the spacings were simply the result of a single uniform density component (Figure 3). In order to compare these models, we maximized the likelihood under each hypothesis using expectation-maximization (EM) [18] (see Materials and methods, below) and computed the likelihood ratio statistic:

Λ=2log⁡[p(data|H1)p(data|H0)],
 MathType@MTEF@5@5@+=feaafiart1ev1aaatCvAUfeBSjuyZL2yd9gzLbvyNv2Caerbhv2BYDwAHbqedmvETj2BSbqee0evGueE0jxyaibaiKI8=vI8tuQ8FMI8Gi=hEeeu0xXdbba9frFj0=OqFfea0dXdd9vqai=hGuQ8kuc9pgc9s8qqaq=dirpe0xb9q8qiLsFr0=vr0=vr0dc8meaabaqaciGacaGaaeqabaqadeqadaaakeaacqqHBoatcqGH9aqpcaaIYaGaciiBaiaac+gacaGGNbWaamWaaeaadaWcaaqaaiaadchacaGGOaGaamizaiaadggacaWG0bGaamyyaiaacYhacaWGibWaaSbaaSqaaiaaigdaaeqaaOGaaiykaaqaaiaadchacaGGOaGaamizaiaadggacaWG0bGaamyyaiaacYhacaWGibWaaSbaaSqaaiaaicdaaeqaaOGaaiykaaaaaiaawUfacaGLDbaacaGGSaaaaa@4D59@

Where *data *represents the observed spacings and corresponding (strong or weak) consensus matches. Because *H*_*0 *_corresponds to the case of *H*_*1 *_with the parameters of the two components constrained to be equal, we expect the LRT statistic (Λ) to be χ^2 ^distributed with three degrees of freedom (see Materials and methods, below).

We therefore computed the *P *values for the LRT on the known targets, the set of 'unbiased positives', the remaining randomly chosen proteins that were found not to be targets of Cdk1p in the assay [11] (henceforth referred to as 'unbiased negatives'; see Table 1), and the 'known' targets using the scrambled consensus sequences (Table 2). Consistent with the model that *bona fide *targets contain clusters of consensus matches, rather than a simple overall enrichment, we could reject the overall enrichment hypothesis in the first two tests (*P *= 1.2 × 10^-9 ^and *P *= 1.6 × 10^-4^, respectively), but not in the latter two negative controls (*P *= 0.13 and *P *= 0.15, respectively; see Table 2).

### Methods to detect clustering in individual proteins

Having established statistical enrichment and tendency for consensus matches to cluster in the primary sequence of *bona fide *CDK targets, we developed a method to predict CDK targets based on these properties. For each protein, we sought to compare the likelihood of the observed matches and spacings given the genome frequencies (*H*_*bg*_) with the likelihood under a two-component model (*H*_*c*_), in which one component is the background genome model and the other is high-frequency 'cluster' component whose parameters are estimated from the protein of interest. This suggests ranking genes according to the following:

S=log⁡[p(data|Hc)p(data|Hbg)]
 MathType@MTEF@5@5@+=feaafiart1ev1aaatCvAUfeBSjuyZL2yd9gzLbvyNv2Caerbhv2BYDwAHbqedmvETj2BSbqee0evGueE0jxyaibaiKI8=vI8tuQ8FMI8Gi=hEeeu0xXdbba9frFj0=OqFfea0dXdd9vqai=hGuQ8kuc9pgc9s8qqaq=dirpe0xb9q8qiLsFr0=vr0=vr0dc8meaabaqaciGacaGaaeqabaqadeqadaaakeaacaWGtbGaeyypa0JaciiBaiaac+gacaGGNbWaamWaaeaadaWcaaqaaiaadchacaGGOaGaamizaiaadggacaWG0bGaamyyaiaacYhacaWGibWaaSbaaSqaaiaadogaaeqaaOGaaiykaaqaaiaadchacaGGOaGaamizaiaadggacaWG0bGaamyyaiaacYhacaWGibWaaSbaaSqaaiaadkgacaWGNbaabeaakiaacMcaaaaacaGLBbGaayzxaaaaaa@4C96@

Because the weak CDK consensus matches the specificity of any proline-directed kinase, we were concerned that some of our predictions would not be specific to CDKs. In order to rule out these cases, we defined a 'nonspecific' model (*H*_*ns*_) as above, except that the frequency of strong matches in the high-frequency 'cluster' component was constrained to be less than or equal to the background genome frequency. We optimized the likelihood under each of these models for each protein (see Materials and methods, below) and ranked them by a classifier assuming uniform 'priors' over the various models:

SLR=log⁡[p(data|Hc)p(data|Hbg)+p(data|Hns)]
 MathType@MTEF@5@5@+=feaafiart1ev1aaatCvAUfeBSjuyZL2yd9gzLbvyNv2Caerbhv2BYDwAHbqedmvETj2BSbqee0evGueE0jxyaibaiKI8=vI8tuQ8FMI8Gi=hEeeu0xXdbba9frFj0=OqFfea0dXdd9vqai=hGuQ8kuc9pgc9s8qqaq=dirpe0xb9q8qiLsFr0=vr0=vr0dc8meaabaqaciGacaGaaeqabaqadeqadaaakeaacaWGtbWaaSbaaSqaaiaadYeacaWGsbaabeaakiabg2da9iGacYgacaGGVbGaai4zamaadmaabaWaaSaaaeaacaWGWbGaaiikaiaadsgacaWGHbGaamiDaiaadggacaGG8bGaamisamaaBaaaleaacaWGJbaabeaakiaacMcaaeaacaWGWbGaaiikaiaadsgacaWGHbGaamiDaiaadggacaGG8bGaamisamaaBaaaleaacaWGIbGaam4zaaqabaGccaGGPaGaey4kaSIaamiCaiaacIcacaWGKbGaamyyaiaadshacaWGHbGaaiiFaiaadIeadaWgaaWcbaGaamOBaiaadohaaeqaaOGaaiykaaaaaiaawUfacaGLDbaaaaa@5940@

This will assign lower scores to proteins that have clusters of only weak consensus matches. Cdc6p (Figure 1a), for example, has *S*_*LR *_= 7.28, and ranks 22^nd ^in the genome.

### Identifying optimal clusters

The mixture models we have employed thus far do not assume that the closely spaced matches fall in a single contiguous region of the primary sequence. We considered this appropriate because residues may be adjacent in the structure of the protein but not in the primary sequence. Nevertheless, we were also interested in identifying the continuous subregions of proteins that contain high densities of matches, such as the amino-terminal domain of Cdc6p (Figure 1b). We therefore also developed a method to identify the most significant 'cluster' of matches within each protein. While *S*_*LR *_(described above) measures 'clustering' in the whole protein, this method allows identification of a single optimal 'cluster'. This represents an alternate strategy to predict proteins that contain clusters of consensus matches - by explicitly identifying the clusters. We note that this does assume that the clustered matches occur in a contiguous region, and therefore, for example, in the case of Cdc6p (Figure 1a) the carboxyl-terminal matches would not contribute to the score.

To find optimal clusters, we counted the number of matches (*n*) to the strong (*s*) or weak (*w*) consensus in each possible subregion of the protein of length *l*. We then computed the probability of observing as many matches or more of each type using the binomial distribution, and combined these *P *values by multiplying them together by assigning a *P *value to their product using the Q-fast algorithm [19]. We note that the subregion with the maximal score will begin and end with a match. There are therefore only *N*(*N *- 1)/2 possible clusters to try, where *N *(= *n*_*s *_+ *n*_*w*_) is the total number of matches in the entire protein. This means that proteins with many matches have more chances to obtain a high scoring cluster. We therefore correct for the total number of clusters searched by multiplying the *P *value by this factor (a Bonferoni multiple testing correction). Thus, we define the following:

SBN=−log⁡[N(N−1)2×Q[p(≥ns|l,fsb)×p(≥nw|l,fwb)]],
 MathType@MTEF@5@5@+=feaafiart1ev1aaatCvAUfeBSjuyZL2yd9gzLbvyNv2Caerbhv2BYDwAHbqedmvETj2BSbqee0evGueE0jxyaibaiKI8=vI8tuQ8FMI8Gi=hEeeu0xXdbba9frFj0=OqFfea0dXdd9vqai=hGuQ8kuc9pgc9s8qqaq=dirpe0xb9q8qiLsFr0=vr0=vr0dc8meaabaqaciGacaGaaeqabaqadeqadaaakeaacaWGtbWaaSbaaSqaaiaadkeacaWGobaabeaakiabg2da9iabgkHiTiGacYgacaGGVbGaai4zamaadmaabaWaaSaaaeaacaWGobGaaiikaiaad6eacqGHsislcaaIXaGaaiykaaqaaiaaikdaaaGaey41aqRaamyuamaadmaabaGaamiCaiaacIcacqGHLjYScaWGUbWaaSbaaSqaaiaadohaaeqaaOGaaiiFaiaadYgacaGGSaGaamOzamaaBaaaleaacaWGZbGaamOyaaqabaGccaGGPaGaey41aqRaamiCaiaacIcacqGHLjYScaWGUbWaaSbaaSqaaiaadEhaaeqaaOGaaiiFaiaadYgacaGGSaGaamOzamaaBaaaleaacaWG3bGaamOyaaqabaGccaGGPaaacaGLBbGaayzxaaaacaGLBbGaayzxaaGaaiilaaaa@6157@

where *Q *[...] is the Q-fast algorithm, *p*(≥ *x *| *l*, *f*) is the binomial probability of observing *x *or more in *l *tries when the per try probability is *f*, and *f*_*sb *_and *f*_*wb *_are the per residue probabilities of observing strong and weak matches, respectively, in the genome. Once again we were concerned about the possibility of nonspecific clusters and therefore, when using *S*_*BN *_to predict CDK targets, we imposed the following heuristic; to be considered, subregions must contain at least one match to the strong consensus per 100 residues. For example, in the case of Cdc6p, this optimal cluster corresponds to the amino-terminal domain (Figure 1b, bold residues) and has *S*_*BN *_= 8.38, ranking 61st in the genome.

### Assessing the classifiers

In order to assess whether these classifiers were capturing useful information about the recognition of substrates by CDKs, we computed the scores described above for each protein in *S. cerevisiae *and compared them to the 'phosphorylation scores' reported for the 695 *S. cerevisiae *proteins tested in the high-throughput Cdk1p assay [11] (Table 1). These proteins tested in that study fall into three groups: 198 randomly chosen proteins (containing the 'unbiased positives' and 'unbiased negatives' described above, henceforth referred to as 'unbiased'), all 385 *S. cerevisiae *proteins that contain two or more matches to the strong CDK consensus (henceforth '2+'), and finally 137 proteins that contain one match to the strong consensus, and exhibit cell cycle transcript regulation (henceforth '1cc'). We note that although the last two groups were biased in different ways, as long as we treat them separately (condition on the bias) the proteins in each group can be treated as identical and independently distributed.

In the 'unbiased' and '2+' groups, we found a highly significant correlation (R > 0.3, *P *< 10^-10^) between the phosphorylation score in the assay and both of the cluster-based scores described above (Table 3), such that proteins with higher scoring cluster are more likely to have high scores in the kinase assay.

Because in many cases we noted that the clusters seemed to occur near the carboxyl- or amino-terminus of the proteins (as in the case of the Cdc6p amino-terminal domain; Figure 1), we computed the relative 'position' of the optimal cluster, where 0.5 is the midpoint of the protein and 0 is either terminus (see Materials and methods, below). Interestingly, we found that the position was negatively correlated (R < -0.2, *P *< 0.01), with the results of the kinase assay in the same two groups of targets, such that proteins with clusters near their termini were more likely to be positive in the assay. It has also been noted that phosphorylation sites tend to fall in disordered or unfolded regions of proteins [20]. Consistent with this, we found a significant correlation (R ≤ -0.19, *P *< 0.01) between the 'foldedness' [21,22] of the cluster and the score in the kinase assay, such that proteins containing clusters of matches in unfolded regions were more likely to be *bona fide *substrates. In order to verify that these factors were independently correlated with the results of the assay (and not simply correlated with each other), we fit linear models of the likelihood ratio score, position and 'foldedness', and found that they all contributed significantly (*P *< 0.02; Table 3).

### Predicting CDK substrates based on clustering of consensus matches

The correlations we observed suggested that clustering of consensus matches could be used to predict the targets of Cdk1p in *S. cerevisiae*. Taking proteins defined as CDK targets or not in the high-throughput assay [11] as positives and negatives, we computed receiver operating characteristic (ROC) curves for the three groups of proteins tested in the assay.

**Figure 4:**
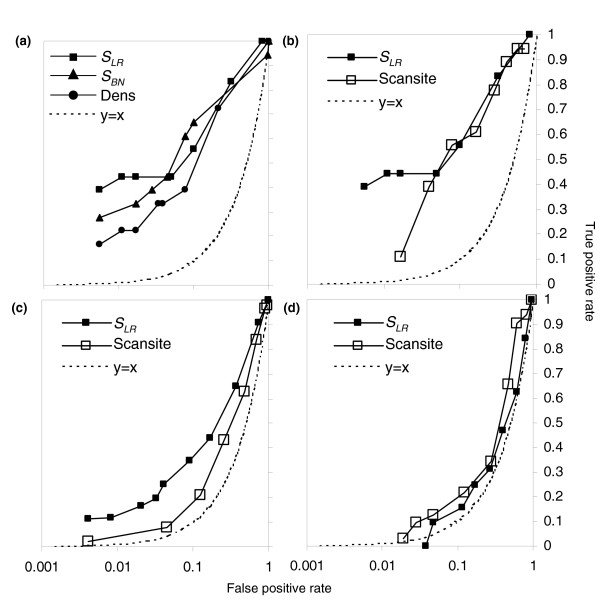
ROC curves for prediction of CDK substrate proteins. **(a) **Comparison of classifiers suggests that cluster based methods S_*LR *_and S_*BN *_(filled squares and triangles, respectively) perform better than the density of strong matches (filled circles). **(b-d) **comparison of cluster-based method S_*LR *_(filled squares) with Scansite, a matrix-based method (unfilled squares). See text for details. Plotted is the fraction of positives versus the fraction of negatives passing as the threshold is varied in the three datasets a, b ('unbiased' proteins, which were randomly chosen), c ('2+' proteins, which contain two or more matches to the strong CDK consensus), and d ('1cc' proteins containing one match to the strong CDK consensus and whose transcripts exhibit cell-cycle regulation). Note that the unlike conventional ROC curves, we plot the false-positive rate on a log scale, such that the expectation for a random predictor no longer falls on the diagonal. The expectation for a random predictor is indicated in each panel by the dotted trace. CDK, cyclin-dependent kinase; ROC, receiver operating characteristic.

First, we compared the two classifiers described above to simply classifying based on the density of strong CDK matches in the protein. We found that although all were strong classifiers in the 'unbiased' set, the cluster-based methods performed better than a simple density (Figure 4a). In the low false-positive range, which is of most relevance to protein database searches, the score based on the likelihood ratio (*S*_*LR*_) seemed most effective. We also compared the methods on the '2+' set and found similar results (data not shown). We therefore used *S*_*LR *_for subsequent analyses.

We next compared the predictive power of the cluster-based classifier (*S*_*LR*_) with that of a specificity matrix-based approach (Scansite [23]), and used the score of the best match to the Cdc2 matrix in each protein (see Materials and methods, below) as the predictor. Both our cluster-based method and the specificity matrix-based method were strong classifiers for the 'unbiased' set (Figure 4b); since most of these proteins contain no matches, many of the negatives can be ruled out simply based on the absence of a match to the consensus. For the '1cc' proteins, neither method has much power (Figure 4d). For the '2+' set (Figure 4c), however, we notice a considerable increase in sensitivity and specificity in the low false-positive region by using our cluster score. In the '2+' group, at false-positive levels near 5%, the matrix-based method performs similar to a random classifier, whereas the cluster-based method retains some power. Because each of these proteins has multiple matches to the consensus, most have high matrix match scores. The proteins in which there are multiple matches that are spatially clustered, however, are more likely to be *bona fide *substrates for Cdk1p. We note that even in this set the overall predictive power is still relatively poor.

**Figure 5:**
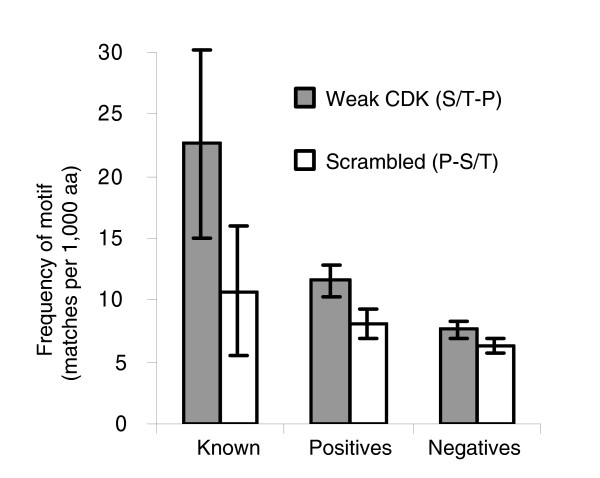
Weak CDK consensus matches co-cluster with strong matches. Gray and unfilled bars indicate frequencies of matches to the weak CDK consensus and to a scrambled version of it within regions identified as optimal clusters based on only strong matches. 'Known' are well characterized CDK substrates, and 'positives' and 'negatives' are proteins scoring greater than and less than 2 in a high-throughput kinase assay, respectively. See text for details. Frequencies are number of matches per 1,000 amino acid (aa) residues. Error bars represent plus or minus two times the standard error. CDK, cyclin-dependent kinase.

An important feature of these cluster based methods is that we can include weak matches to the consensus in our predictor. We found, however, that classifiers based on clustering only of strong matches also performed well (data not shown). In order to confirm that the weak matches were contributing to the clusters, we identified optimal clusters based only on the strong matches using a univariate version of the method described above (*S*_*BN*_). We then compared the density of weak matches in these regions with the density of the scrambled weak consensus. We found enrichment of 2.1-fold and 1.4-fold in the 'known' targets and assay positives (all groups combined), as compared with 1.2-fold in the negatives (all groups combined; Figure 5), indicating that weak matches are preferentially associated with clusters of strong matches. The size of these effects is not great, however, and therefore weak matches may not contribute much to the classification of individual proteins. Nevertheless, this supports the use of both the strong and weak consensus matches in this case, and is consistent with previous reports that weak sites can be important for function [12] (see Discussion, below).

Our aim here was not to explore the properties of these classifiers in detail, but rather to establish the potential of methods that take advantage of the propensity of the CDK motifs to cluster (see Discussion, below).

### Defining a set of proteins containing clusters of CDK consensus sequences

**Figure 6:**
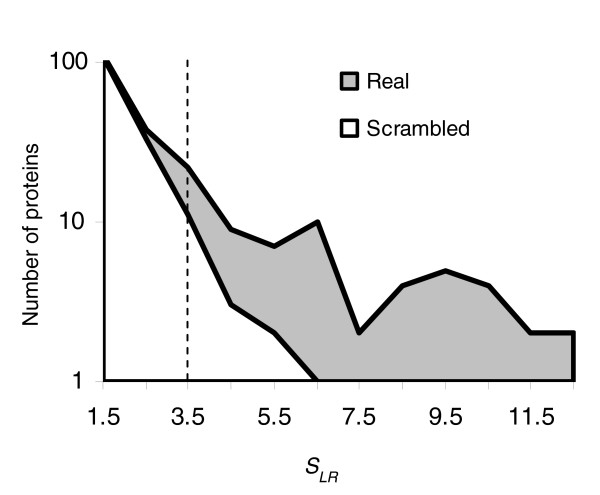
Defining a set of CDK consensus cluster containing proteins. Comparison of the distribution of scores from a search of the *S. cerevisiae *genome using either the real CDK consensus motifs (gray area) or scrambled versions (unfilled area) suggests a threshold of 3.5 (dotted line). CDK, cyclin-dependent kinase.

Taken together, these results suggest that not all Cdk1p targets in *S. cerevisiae *contain clusters of consensus matches, but that there is some subset that can be predicted in this way. In order to estimate the number of CDK consensus cluster containing proteins that can be recognized based on sequence alone, we searched the genome for matches to scrambled versions of the strong and weak CDK consensus (P-R/K-X-S/T and P-S/T, respectively) and compared the distribution of likelihood ratio scores with those obtained using the real consensus sequences. Comparison of these distributions suggests a score threshold of 3.5 (Figure 6). This yields an excess of 50 proteins, because there are 67 proteins above the threshold when the real consensus sequences are used, and 17 when scrambled consensus sequences are used.

Of these 67 top predictions (ranked based only on sequence), 49 were positive in the kinase assay [11] (all groups combined). This indicates at this threshold our cluster-based method yields a positive predictive value (PPV) of 73%, but it includes 18 false positives. Compared with the PPV of 49% (17/35) for the proteins identified by the matrix-based approach (Scansite [23]) at the same false-positive level, our cluster-based approach has significantly greater PPV (*P *= 0.017, by Fisher's exact test), which is consistent with the hypothesis that searching for clusters can strongly identify at least some subset of CDK targets. In order to examine further the properties of the clustered matches in these proteins, we identified the maximal scoring cluster using the method described above (*S*_*BN*_). Consistent with our earlier observations, we found that for 36% (24/67) of these proteins the optimal cluster ended within 5% of the protein's length from either terminus, and that even if we masked the CDK matches, the optimal clusters were on average significantly less 'folded' that the whole proteins (-0.08 versus -0.0002, respectively; *P *< 0.001, by Students' *t*-test).

### Predicting CDK targets among human proteins

Regulation of cell cycle progression by CDKs is thought to be an ancient feature of eukaryotic cells. Indeed, human CDK homologs were first identified based on their ability to rescue yeast mutants [24,25]. We therefore sought to test whether clustering of consensus matches could also be used to predict CDK targets in humans.

**Figure 7:**
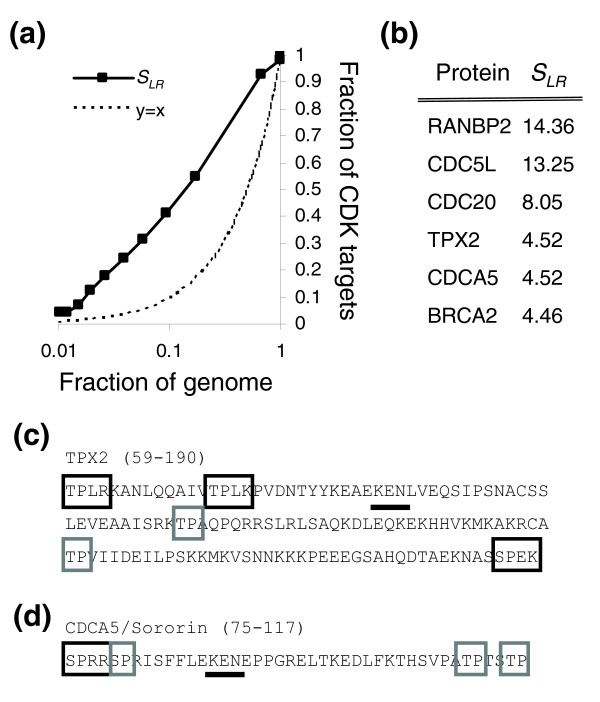
Predicting CDK targets in the human genome. **(a) **The fraction of proteins in known human CDK targets versus the fraction in the human genome (black bar) as the cutoff is varied. **(b) **Genes with clusters scoring more than 3.5 from a list of human cell-cycle genes. See text for details. **(c,d) **The K-E-N box (black underline) degradation signals in TPX2 (panel c) and Sororin (panel d) are found among clustered consensus matches. The entire optimal clusters are not shown. Strong and weak consensus matches are indicated by black and grey boxes, respectively. The regions of the protein shown are indicated in parentheses. CDK, cyclin-dependent kinase.

We found 73 human proteins (see Materials and methods, below) that were listed as CDK, CDK1, or CDK2 targets in the phosphoELM database [26]. Although we do not have a set of negative proteins (as for *S. cerevisiae*), we can still compute an ROC curve by using the fraction of the genome above the threshold as an approximate false-positive rate. In doing so we assume that the fraction of proteins that are targets in the genome is negligible compared with the total number of proteins. This analysis (Figure 7a) suggests that our method has some predictive power at reasonably low false-positive levels; some subset of human CDK targets may also contain clusters of consensus matches and may therefore be predicted using our method.

To predict novel human CDK targets, we obtained a set of 112 human cell cycle genes (see Materials and methods) and identified those containing clustered consensus matches. Of the six proteins in this set with clusters scoring 3.5 or greater (Figure 7b), none were included in the 73 CDK targets in phosphoELM. Of these, BRCA2 was recently shown to be a CDK target [27]. Of the other five, there is already evidence that three (RANBP2, CDC20, and CDC5L) are mitotic phosphoproteins, and there are varying degrees of evidence that they are *bona fide *CDK targets [28-30]. The other two (CDCA5/sororin and TPX2) are both degraded by the anaphase-promoting complex through direct interaction with K-E-N motifs [31,32]. Interestingly, these K-E-N motifs are found among closely spaced CDK consensus matches in these proteins (Figure 7c,d). It is tempting to speculate that their anaphase promoting complex-dependent degradation is regulated through phosphorylation by CDKs, as has been suggested for human CDC6 [33], and that these clusters represent regulatory modules (see Discussion, below). Regardless, that these human cell cycle proteins contain clusters of CDK consensus sequences, and that there is some evidence for CDK phophorylation for four of the six, suggests that cluster-based methods can be used to predict CDK targets among human proteins as well.

### Clusters of consensus matches and cyclin specificity

**Figure 8:**
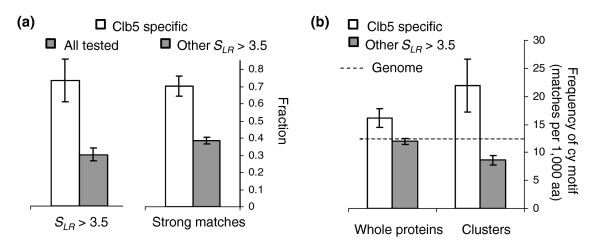
Clustering of CDK consensus matches and cyclin specificity. **(a) **The left side shows that clb5-specific CDK targets (unfilled bar) are more likely to score above the cutoff than other proteins assayed (gray bar), while the right side of panel a shows that clb5-specific CDK targets (unfilled bar) contain a higher proportion of strong matches than do other high-scoring proteins (gray bar). See text for details **(b) **CDK targets specific for clb5 (unfilled bars) contain an excess of matches to the cy motif relative to other high-scoring proteins (gray bars) in the entire protein sequence (left side), but this enrichment is more extreme if only regions containing clustered CDK consensus matches are considered (right side). The dotted line represents the genomic frequency of matches to the cy motif. CDK, cyclin-dependent kinase; aa, amino acids.

CDKs are thought to gain target specificity by pairing with particular cyclins. For example, Cdc6p was found to be a specific target of Cdk1p:Clb5p [34] and contains cyclin specific 'cy' motifs (R/K-X-L [17]) in addition to CDK motifs (Figure 1b, filled bars). We noted that of 14 Cdk1p:Clb5p specific targets identified in a recent study [34], 72% (10) where among our strongest *S. cerevisiae *predictions (*S*_*LR *_> 3.5). Because, of the 143 proteins tested in that study, only 29% (42) were included in this set (*S*_*LR *_> 3.5), 72% represents a highly significant enrichment (*P *< 0.001, Fisher's exact test; Figure 8a, left side). Interestingly, we also found that the clb5 specific proteins above our cutoff contained a higher proportion of strong matches to the CDK consensus; the clb5 specific clusters contained 43 strong and 18 weak matches (70% strong), which is significantly more than in the clusters in the rest of the proteins above the cutoff, where we find 217 strong and 343 weak (39% strong; *P *< 0.001, by Fisher's exact test; Figure 8a, right side). We speculate that this may be related to the lower overall activity of the Cdk1p-Clb5p complex [34].

In order to test directly whether 'cy' motifs were associated with the CDK clusters, we masked out the matches to the CDK consensus and compared the frequency of matches to the cy motif in the clb5 specific proteins with the frequency in the rest of the proteins above the cutoff (Figure 8b). Although the frequency of cy motifs in the entire proteins was significantly greater in the clb5-specific targets than in the other proteins (Figure 8b, left side; *P *= 0.014, by Fisher's exact test), the difference was greater and more significant when we considered only the regions identified as optimal clusters (Figure 8b, right side; *P *< 0.001, by Fisher's exact test). Futhermore, we note that the regions defined as the optimal clusters in the proteins that were not clb5 specific contain fewer matches to this motif than expected based on the genome frequency, perhaps related to the paucity of leucine residues near phosphorylation sites [20]. These findings suggest that cy motifs tend to cluster with CDK motifs in clb5 specific targets. Thus, it may be possible to associate cyclin specificity with a specific composition of motifs, analogous to the 'regulatory codes' that have been proposed for some enhancers of transcription [35] (see Discussion, below).

## Discussion

We divide the discussion into two sections, the first addressing biologic considerations and the second methodology.

### Biology

Several characterized CDK target proteins have multiple consensus phosphorylation sites, often restricted to particular regions of the protein. We confirmed that known *S. cerevisiae *CDK targets are statistically enriched for CDK consensus matches (Figure 2) and that these are closely spaced (clustered) in the linear sequence of these proteins (Figure 3 and Table 2). We showed that spatial clustering is significantly associated with *bona fide *CDK substrate proteins in *S. cerevisiae *(Table 3) and human (Figure 7a), and a search of human cell cycle genes suggested several plausible CDK targets, some of which already have various degrees of supporting evidence (Figure 7b).

Noncoding regulatory DNA elements, such as enhancers (or *cis*-regulatory modules), often contain clusters of binding sites for transcription factors [36,37], and computational methods have been developed to exploit this [38]. In analogy, we suggest that the regions of proteins containing the clusters of CDK consensus matches may be regarded as phospho-regulatory modules. As with *cis*-regulatory modules, they may contain additional regulatory elements, such as the phosphorylation sites of other kinases, localization and degradation signals, and other protein recognition motifs. For example, the amino-terminal domain of *S. cerevisiae *Cdc6 (Figure 1b) contains a cluster of CDK consensus matches, as well as a nuclear localization signal [39].

As an illustration of a potential mechanistic basis for this model, consider the case of clusters of phosphorylation sites proximal to nuclear localization signals (NLSs). Nuclear import is often mediated through the interaction of importins (or karyopherins) with NLSs, which are basic, hydrophilic motifs [40]. The addition of multiple bulky, negatively charged phosphates proximal to these motifs have the potential inhibit their function; indeed, several examples of such inhibitory phosphorylation of nuclear localization signals have been described (for review, see Jans and Hubner [40]), including inhibition of the SV40 and Swi5p NLSs by phosphorylation of partially overlapping or proximal consensus sites by CDKs [41,42] (Figure 1a). We suggest that such local interactions between motifs may be a general mechanism by which regulatory specificity can be achieved.

Additionally, characterized cy motifs, such as the cy motif in Orc6p [43], often occur in proximity to clusters of phosphorylation sites (Figure 1a). We showed that the clusters of CDK matches in clb5-specific CDK targets contained a higher fraction of strong CDK consensus matches and an excess of cy motifs (Figure 8). These observations are consistent with the lower activity of this CDK-cyclin combination [34] and its known dependence on the cy motif for substrate recognition [10]. Although the cy motif is known to interact directly with cyclin, it is not mechanistically obvious why multiple copies of this motif would be proximal to the CDK sites in the linear amino acid sequence. Nevertheless, as has been suggested for control of transcriptional regulation [35], we suggest that these features form a regulatory 'grammar' or 'code' that produces a specific pattern of activity. To test this model further we also examined the order of strong and weak sites in the clusters, but we found no striking patterns (data not shown). We suggest that as more data become available, and computational methods for the prediction of short protein motifs continue to improve, it will be possible to identify increasingly specific combinations of motifs associated with particular patterns of regulation.

It is also important to note that there are additional possible biological explanations for the large numbers of CDK consensus matches, such as precise control of regulatory thresholds [12] or highly cooperative binding [44], and that because there are many determinants of kinase-substrate specificity, clustering of regulatory motifs cannot be expected to reveal all (or any) of the substrates of a particular kinase. The general utility of the methods proposed here will depend on the specific biology of each regulatory system.

Indeed, it is perhaps surprising that the proximity of features of the primary amino acid sequence are predictive at all, because these may not be reflected by the three-dimensional structure of the protein. Interestingly, we found that the clusters of CDK matches tend to fall in relatively disordered regions and near the termini of target proteins (Table 2); perhaps in these regions spatial proximity in the primary sequence is more reflective of proximity *in vivo*. We suggest that this is also consistent with the hypothesis of combinatorial regulation at these regions through multiple motifs; the regions containing these clusters must be accessible to multiple regulatory proteins, and therefore perhaps tend to be flexible, solvent-exposed regions, rather than highly structured domains.

### Methodology

One of the main limitations of this simplistic approach to predicting CDK substrates is that we cannot distinguish between targets of Cdk1p paired with other cyclins, other CDKs, or other regulatory proteins with similar specificity. This means that even our 'false positive' predictions may contain interesting biologic information despite failing to show phosphorylation by Cdk1p-Clb2p in the kinase assay. For example, among the 67 proteins that we predicted to be CDK targets in *S. cerevisiae*, 18 were not found to be targets in the high-thoughput assay [11]. Included in these, we find CDK-cln substrates Ste20p and Whi5p [45,46], as well as Cdc14p phosphatase substrates Sli15p and Cdc15p [47]. These examples further demonstrate that CDK consensus clusters often point to functionally important interactions; identifying sequence features that distinguish these different types of interactions is an area for further research.

Despite its simplicity, spatial clustering represents a potentially powerful new method for computationally identifying a subset of CDK targets. Unlike current methods, which predict individual phosphorylation sites, the statistical methods we have developed identify proteins that exhibit clustering of matches to the CDK consensus. In addition, cluster-based methods can incorporate weak matches to the consensus, and we showed that weak sites tend to co-cluster with strong sites in *bona fide *CDK substrates. Although we have not evaluated whether our method will be applicable to other systems, the methods we have described search for patterns of matches that deviate from a random (geometric) expectation. They can therefore be applied to searches for clusters of any highly degenerate sequence elements, without the need to specify a window size. We suggest, however, that as more is understood about the specific properties of clusters of protein motifs, it will be possible to use more powerful statistical methods to search for particular patterns and combinations of motifs.

A further possible application of spatial clustering of consensus motifs is to identify the functionally important residues in CDK targets. For example, in Cdc6p (Figure 1), the 'clustered' matches correspond to the amino-terminal domain, which is phosphorylated and bound directly by CDKs [14]. In long proteins where there are large numbers of consensus matches, we speculate that those in clusters are more likely to represent the critical phosphorylation sites. Finally, we suggest that cluster-based methods could be used in combination with the sophisticated specificity-based methods that are currently widely used [6], or combined with other evidence, such as structural properties [20], mass spectrometry data [48], or evolutionary conservation [49]. Indeed, given the availability of genome sequences, comparative analysis of CDK clustering should yield insights into the evolution of post-translational regulation.

## Materials and methods

### Genes and proteins

For *S. cerevisiae*, we obtained the protein translations from SGD [50]. For human, for each gene in Ensembl [51] (release 36i) we took the longest translation, which yielded 22,217 genes.

We obtained the human CDK targets from the phosphoELM database [26] by parsing the webpage CDK, CDK1, or CDK2 to obtain uniprot IDs and then looking these up in Ensembl. Thus, the 73 CDK targets may not include all of the CDK targets in phosphoELM. A list of 112 human genes with described cell cycle phenotypes in human cells was obtained from the Mitocheck database [52]. The 73 known human CDK targets and 112 human cell cycle genes are available in Additional data file 1.

### Multivariate geometric models

For a given protein sequence, we imagine counting the residues after we see a match until we observe another match to the consensus. Once one is observed, we record the number of residues and the type of match (strong or weak) that was observed. We denote the match as a vector *X*, such that a strong match is represented as *X *= (1,0) and a weak match as (0,1). If matches occur independently, then the probability of any particular match is given by the following.

p(X,l)=(1−ϕ)l−1∏mfmXm,
 MathType@MTEF@5@5@+=feaafiart1ev1aaatCvAUfeBSjuyZL2yd9gzLbvyNv2Caerbhv2BYDwAHbqedmvETj2BSbqee0evGueE0jxyaibaiKI8=vI8tuQ8FMI8Gi=hEeeu0xXdbba9frFj0=OqFfea0dXdd9vqai=hGuQ8kuc9pgc9s8qqaq=dirpe0xb9q8qiLsFr0=vr0=vr0dc8meaabaqaciGacaGaaeqabaqadeqadaaakeaacaWGWbGaaiikaiaadIfacaGGSaGaamiBaiaacMcacqGH9aqpcaGGOaGaaGymaiabgkHiTiabew9aQjaacMcadaahaaWcbeqaaiaadYgacqGHsislcaaIXaaaaOWaaebuaeaacaWGMbWaa0baaSqaaiaad2gaaeaacaWGybWaaSbaaWqaaiaad2gaaeqaaaaaaSqaaiaad2gaaeqaniabg+GivdGccaGGSaaaaa@4860@

Where *m *indexes the types of matches (strong or weak), *f*_*m *_is the per residue probability of observing a match of type *m*, ϕ=∑mfm
 MathType@MTEF@5@5@+=feaafiart1ev1aaatCvAUfeBSjuyZL2yd9gzLbvyNv2Caerbhv2BYDwAHbqedmvETj2BSbqee0evGueE0jxyaibaiKI8=vI8tuQ8FMI8Gi=hEeeu0xXdbba9frFj0=OqFfea0dXdd9vqai=hGuQ8kuc9pgc9s8qqaq=dirpe0xb9q8qiLsFr0=vr0=vr0dc8meaabaqaciGacaGaaeqabaqadeqadaaakeaacqaHvpGAcqGH9aqpdaaeqbqaaiaadAgadaWgaaWcbaGaamyBaaqabaaabaGaamyBaaqab0GaeyyeIuoaaaa@3B12@ is the probability of any match, and *l *is the total number of residues observed. We note that this defines a multivariate geometric distribution and we can use the geometric series (∑k=0∞rk=11−r
 MathType@MTEF@5@5@+=feaafiart1ev1aaatCvAUfeBSjuyZL2yd9gzLbvyNv2Caerbhv2BYDwAHbqedmvETj2BSbqee0evGueE0jxyaibaiKI8=vI8tuQ8FMI8Gi=hEeeu0xXdbba9frFj0=OqFfea0dXdd9vqai=hGuQ8kuc9pgc9s8qqaq=dirpe0xb9q8qiLsFr0=vr0=vr0dc8meaabaqaciGacaGaaeqabaqadeqadaaakeaadaaeWbqaaiaadkhadaahaaWcbeqaaiaadUgaaaaabaGaam4Aaiabg2da9iaaicdaaeaacqGHEisPa0GaeyyeIuoakiabg2da9maalaaabaGaaGymaaqaaiaaigdacqGHsislcaWGYbaaaaaa@4013@) to show the following:

p(X)=∑l=1∞p(X,l)=1ϕ∏mfmXm
 MathType@MTEF@5@5@+=feaafiart1ev1aaatCvAUfeBSjuyZL2yd9gzLbvyNv2Caerbhv2BYDwAHbqedmvETj2BSbqee0evGueE0jxyaibaiKI8=vI8tuQ8FMI8Gi=hEeeu0xXdbba9frFj0=OqFfea0dXdd9vqai=hGuQ8kuc9pgc9s8qqaq=dirpe0xb9q8qiLsFr0=vr0=vr0dc8meaabaqaciGacaGaaeqabaqadeqadaaakeaacaWGWbGaaiikaiaadIfacaGGPaGaeyypa0ZaaabCaeaacaWGWbGaaiikaiaadIfacaGGSaGaamiBaiaacMcaaSqaaiaadYgacqGH9aqpcaaIXaaabaGaeyOhIukaniabggHiLdGccqGH9aqpdaWcaaqaaiaaigdaaeaacqaHvpGAaaWaaebuaeaacaWGMbWaa0baaSqaaiaad2gaaeaacaWGybWaaSbaaWqaaiaad2gaaeqaaaaaaSqaaiaad2gaaeqaniabg+Givdaaaa@4D40@

This, as expected, is *f*_*s*_/(*f*_*s*_*+ f*_*w*_) or *f*_*w*_/(*f*_*s*_*+ f*_*w*_), for a strong or weak match, respectively. The distribution of spacings, on the other hand, is given by another geometric distribution:

p(l)=∑Xp(X,l)=∑m(1−ϕ)l−1fm=(1−ϕ)l−1ϕ
 MathType@MTEF@5@5@+=feaafiart1ev1aaatCvAUfeBSjuyZL2yd9gzLbvyNv2Caerbhv2BYDwAHbqedmvETj2BSbqee0evGueE0jxyaibaiKI8=vI8tuQ8FMI8Gi=hEeeu0xXdbba9frFj0=OqFfea0dXdd9vqai=hGuQ8kuc9pgc9s8qqaq=dirpe0xb9q8qiLsFr0=vr0=vr0dc8meaabaqaciGacaGaaeqabaqadeqadaaakeaacaWGWbGaaiikaiaadYgacaGGPaGaeyypa0ZaaabuaeaacaWGWbGaaiikaiaadIfacaGGSaGaamiBaiaacMcaaSqaaiaadIfaaeqaniabggHiLdGccqGH9aqpdaaeqbqaaiaacIcacaaIXaGaeyOeI0Iaeqy1dOMaaiykamaaCaaaleqabaGaamiBaiabgkHiTiaaigdaaaGccaWGMbWaaSbaaSqaaiaad2gaaeqaaaqaaiaad2gaaeqaniabggHiLdGccqGH9aqpcaGGOaGaaGymaiabgkHiTiabew9aQjaacMcadaahaaWcbeqaaiaadYgacqGHsislcaaIXaaaaOGaeqy1dOgaaa@5777@

In order to test the hypothesis that the matches are clustered relative to what would be expected under a single frequency for each class of match, we defined a two-component mixture of multivariate geometric distributions, such that:

p(X,l)=π(1−ϕ1)l−1∏mf1mXm+(1−π)(1−ϕ2)l−1∏mf2mXm,
 MathType@MTEF@5@5@+=feaafiart1ev1aaatCvAUfeBSjuyZL2yd9gzLbvyNv2Caerbhv2BYDwAHbqedmvETj2BSbqee0evGueE0jxyaibaiKI8=vI8tuQ8FMI8Gi=hEeeu0xXdbba9frFj0=OqFfea0dXdd9vqai=hGuQ8kuc9pgc9s8qqaq=dirpe0xb9q8qiLsFr0=vr0=vr0dc8meaabaqaciGacaGaaeqabaqadeqadaaakeaacaWGWbGaaiikaiaadIfacaGGSaGaamiBaiaacMcacqGH9aqpcqaHapaCcaGGOaGaaGymaiabgkHiTiabew9aQnaaBaaaleaacaaIXaaabeaakiaacMcadaahaaWcbeqaaiaadYgacqGHsislcaaIXaaaaOWaaebuaeaacaWGMbWaa0baaSqaaiaaigdacaWGTbaabaGaamiwamaaBaaameaacaWGTbaabeaaaaaaleaacaWGTbaabeqdcqGHpis1aOGaey4kaSIaaiikaiaaigdacqGHsislcqaHapaCcaGGPaGaaiikaiaaigdacqGHsislcqaHvpGAdaWgaaWcbaGaaGOmaaqabaGccaGGPaWaaWbaaSqabeaacaWGSbGaeyOeI0IaaGymaaaakmaarafabaGaamOzamaaDaaaleaacaaIYaGaamyBaaqaaiaadIfadaWgaaadbaGaamyBaaqabaaaaaWcbaGaamyBaaqab0Gaey4dIunakiaacYcaaaa@61C8@

where *f*_*1 *_and *f*_2 _are the parameters for the two components, *ϕ *_*1 *_and *ϕ *_2 _are their sums, and *π *is a mixing parameter or prior probability on which component will be observed. The likelihood of the data under this model is simply the product of terms of this form over all of the observed matches and spacings. In order to obtain maximum likelihood estimates of these parameters, we derived EM [18] update equations as follows. The expected complete log-likelihood of the data is:

〈log⁡[p(data|f,π)]〉=∑i∑c〈Zci〉[log⁡πc+(li−1)log⁡(1−ϕc)+∑mXmilog⁡fcm]
 MathType@MTEF@5@5@+=feaafiart1ev1aaatCvAUfeBSjuyZL2yd9gzLbvyNv2Caerbhv2BYDwAHbqedmvETj2BSbqee0evGueE0jxyaibaiKI8=vI8tuQ8FMI8Gi=hEeeu0xXdbba9frFj0=OqFfea0dXdd9vqai=hGuQ8kuc9pgc9s8qqaq=dirpe0xb9q8qiLsFr0=vr0=vr0dc8meaabaqaciGacaGaaeqabaqadeqadaaakeaadaaadaqaaiGacYgacaGGVbGaai4zamaadmaabaGaamiCaiaacIcacaWGKbGaamyyaiaadshacaWGHbGaaiiFaiaadAgacaGGSaGaeqiWdaNaaiykaaGaay5waiaaw2faaaGaayzkJiaawQYiaiabg2da9maaqafabaWaaabuaeaadaaadaqaaiaadQfadaWgaaWcbaGaam4yaiaadMgaaeqaaaGccaGLPmIaayPkJaaaleaacaWGJbaabeqdcqGHris5aaWcbaGaamyAaaqab0GaeyyeIuoakmaadmaabaGaciiBaiaac+gacaGGNbGaeqiWda3aaSbaaSqaaiaadogaaeqaaOGaey4kaSIaaiikaiaadYgadaWgaaWcbaGaamyAaaqabaGccqGHsislcaaIXaGaaiykaiGacYgacaGGVbGaai4zaiaacIcacaaIXaGaeyOeI0Iaeqy1dO2aaSbaaSqaaiaadogaaeqaaOGaaiykaiabgUcaRmaaqafabaGaamiwamaaBaaaleaacaWGTbGaamyAaaqabaGcciGGSbGaai4BaiaacEgacaWGMbWaaSbaaSqaaiaadogacaWGTbaabeaaaeaacaWGTbaabeqdcqGHris5aaGccaGLBbGaayzxaaaaaa@72F4@

Where *i *indexes the observation, *c *indexes the two components, angled brackets indicate expectations, and *Z*_*ci *_is an unobserved indicator variable that denotes - for each observed match and spacing - which component of the mixture it was drawn from. The maximization step entails setting the derivatives with respect to the parameters to zero, under the constraint that ∑cπc=1
 MathType@MTEF@5@5@+=feaafiart1ev1aaatCvAUfeBSjuyZL2yd9gzLbvyNv2Caerbhv2BYDwAHbqedmvETj2BSbqee0evGueE0jxyaibaiKI8=vI8tuQ8FMI8Gi=hEeeu0xXdbba9frFj0=OqFfea0dXdd9vqai=hGuQ8kuc9pgc9s8qqaq=dirpe0xb9q8qiLsFr0=vr0=vr0dc8meaabaqaciGacaGaaeqabaqadeqadaaakeaadaaeqbqaaiabec8aWnaaBaaaleaacaWGJbaabeaakiabg2da9iaaigdaaSqaaiaadogaaeqaniabggHiLdaaaa@3AD4@. This yields the following:

fcm=∑i〈Zci〉Xmi∑i〈Zci〉li and πc=1N∑i〈Zci〉,
 MathType@MTEF@5@5@+=feaafiart1ev1aaatCvAUfeBSjuyZL2yd9gzLbvyNv2Caerbhv2BYDwAHbqedmvETj2BSbqee0evGueE0jxyaibaiKI8=vI8tuQ8FMI8Gi=hEeeu0xXdbba9frFj0=OqFfea0dXdd9vqai=hGuQ8kuc9pgc9s8qqaq=dirpe0xb9q8qiLsFr0=vr0=vr0dc8meaabaqaciGacaGaaeqabaqadeqadaaakeaacaWGMbWaaSbaaSqaaiaadogacaWGTbaabeaakiabg2da9maalaaabaWaaabuaeaadaaadaqaaiaadQfadaWgaaWcbaGaam4yaiaadMgaaeqaaaGccaGLPmIaayPkJaGaamiwamaaBaaaleaacaWGTbGaamyAaaqabaaabaGaamyAaaqab0GaeyyeIuoaaOqaamaaqafabaWaaaWaaeaacaWGAbWaaSbaaSqaaiaadogacaWGPbaabeaaaOGaayzkJiaawQYiaiaadYgadaWgaaWcbaGaamyAaaqabaaabaGaamyAaaqab0GaeyyeIuoaaaGccaqGGaGaaeyyaiaab6gacaqGKbGaaeiiaiabec8aWnaaBaaaleaacaWGJbaabeaakiabg2da9maalaaabaGaaGymaaqaaiaad6eaaaWaaabuaeaadaaadaqaaiaadQfadaWgaaWcbaGaam4yaiaadMgaaeqaaaGccaGLPmIaayPkJaaaleaacaWGPbaabeqdcqGHris5aOGaaiilaaaa@5DCF@

where *N *is the total number of matches in the dataset respectively. At the expectation step we use Bayes' theorem to calculate the following, using the estimates of the parameters (*f*, *π*) from the previous iteration:

〈Zci〉=p(Zci=1|Xi,li)=πcp(Xi,li|fc)∑dπdp(Xi,li|fd),
 MathType@MTEF@5@5@+=feaafiart1ev1aaatCvAUfeBSjuyZL2yd9gzLbvyNv2Caerbhv2BYDwAHbqedmvETj2BSbqee0evGueE0jxyaibaiKI8=vI8tuQ8FMI8Gi=hEeeu0xXdbba9frFj0=OqFfea0dXdd9vqai=hGuQ8kuc9pgc9s8qqaq=dirpe0xb9q8qiLsFr0=vr0=vr0dc8meaabaqaciGacaGaaeqabaqadeqadaaakeaadaaadaqaaiaadQfadaWgaaWcbaGaam4yaiaadMgaaeqaaaGccaGLPmIaayPkJaGaeyypa0JaamiCaiaacIcacaWGAbWaaSbaaSqaaiaadogacaWGPbaabeaakiabg2da9iaaigdacaGG8bGaamiwamaaBaaaleaacaWGPbaabeaakiaacYcacaWGSbWaaSbaaSqaaiaadMgaaeqaaOGaaiykaiabg2da9maalaaabaGaeqiWda3aaSbaaSqaaiaadogaaeqaaOGaamiCaiaacIcacaWGybWaaSbaaSqaaiaadMgaaeqaaOGaaiilaiaadYgadaWgaaWcbaGaamyAaaqabaGccaGG8bGaamOzamaaBaaaleaacaWGJbaabeaakiaacMcaaeaadaaeqbqaaiabec8aWnaaBaaaleaacaWGKbaabeaakiaadchacaGGOaGaamiwamaaBaaaleaacaWGPbaabeaakiaacYcacaWGSbWaaSbaaSqaaiaadMgaaeqaaOGaaiiFaiaadAgadaWgaaWcbaGaamizaaqabaGccaGGPaaaleaacaWGKbaabeqdcqGHris5aaaakiaacYcaaaa@647C@

We used an implementation of this algorithm to maximize the likelihood and obtain estimates of the parameters. For a set of input matches (characterized by spacing, *l*_*i*_, and type, *X*_*i*_) at convergence, the algorithm produces a set of maximum likelihood estimates of the parameters (*f*, *π*) with which we compute the maximum value of the likelihood. In order to verify that the EM optimization was reliably finding the maximum in the likelihood (it is only guaranteed to give a local maximum), we re-ran the fit using five random sets of initial parameters.

In practice, protein sequences deviate from these geometric models in that they do not in general end with a match to the motif. In order to account correctly for the remaining residues after the final match, we combine them with the residues before the first match. This also ensures that a given set of matches has the same probability regardless of where it occurs in the protein (relative to the start). Another technical issue with the application of geometric models to proteins is that the decision to begin 'counting' the residues from the amino-terminus or 'left' end is arbitrary; we could equally well have started from the carboxyl-terminus or 'right' end. We confirmed that this makes little difference; counting from 'right' to 'left' gave qualitatively very similar results.

To use these geometric models for hypothesis testing we proceed as follows. The single-component multivariate geometric has two parameters (the densities of strong and weak matches, *f*), whereas the two-component mixture has five (two sets of densities, *f*_*1 *_and *f*_2_, and a mixing parameter *π*). We note that these models are nested; the single-component model (*H*_*0*_) is a two-component mixture where the parameters for the two components are constrained to be equal (*f*_*1 *_= *f*_2_). Because the likelihood in the single-component case (*H*_*0*_) is independent of the mixing parameter, *π*, there is a three-parameter difference between the two hypotheses. We therefore expect the distribution of the likelihood ratio statistic to be χ^2 ^with three degrees of freedom [53]. To verify that the distribution of the likelihood ratio statistic was indeed χ^2 ^with three degrees of freedom, we randomly permuted the positions of the consensus matches in the 'known' set 100 times, and computed the likelihood ratio statistic for the comparison of the two models; we found reasonable agreement with expectation (data not shown).

We compute *S*_*LR *_for each protein as follows. For each protein, we obtain the set of matches (their positions and type, strong or weak) and compute the likelihood under the following: *H*_*bg *_(assuming the matches were randomly drawn from the genome frequencies); *H*_*c *_(fitting the mixture using the EM algorithm described above, but keeping the background component set to the genome frequencies); or *H*_*ns *_(as for *H*_*c*_, but additionally constraining the frequency of strong matches in the cluster component to be less than or equal to the background frequency). We combine these likelihoods as is given in Results (above). As before, we run the EM with five random starting points for each protein.

### Position and foldedness of maximal cluster

We identified the optimal cluster using *S*_*BN *_as described above. To compute the position of the cluster, we calculated the distance between the start of the protein and the start of the cluster, and between the end of the cluster and the end of the protein. We then took the minimum of these divided by the length of the protein to be the position. We computed the foldedness as *I*_*f *_= 2.785 × *H *- |*R*| - 1.51 [21,22], where *H *is the average hydropathy [54] per residue and |*R*| is the absolute value of the charge (at pH 7.0) per residue in the cluster.

### Scansite searches

We submitted the yeast protein sequences to the batch Scansite [55] using low stringency, which yielded 12,134 Cdc2 matches in 4,048 of the 5,889 yeast proteins. We then took the best (lowest score) for each of those proteins.

## Additional data files

The following additional data are available with the online version of this paper. Additional data file 1 contains the *S. cerevisiae *proteins with *S*_*LR *_above 3.5, and the human CDK targets and cell-cycle proteins with associated *S*_*LR *_scores. Scripts to calculate *S*_*LR *_and *S*_*BN *_are available on AMM's website [56].

## Supplementary Material

Additional data file 1 This document contains the *Saccharomyces cerevisiae *proteins with *S*_*LR *_greater than 3.5, and the human CDK targets and cell cycle proteins with associated *S*_*LR *_scores. Click here for file

## Figures and Tables

**Table 1:** CDK target sets used in this study

Set	Ascertainment	Total	Definition of target	Positives
'Known'	Unknown, complex	12	Low-throughput experimental characterization	12
'Unbiased'	randomly chosen proteins	198	Score > 2 in high-throughput assay	18
'2+'	All *S. cerevisiae *proteins containing two or more matches to the 'strong' CDK consensus	385	Score > 2 in high-throughput assay	143
'1cc'	All *S. cerevisiae *proteins containing one match to the 'strong' CDK consensus and exhibiting cell cycle regulated transcription	137	Score > 2 in high-throughput assay	32

Four cyclin-dependent kinase (CDK) target sets from *Saccharomyces cerevisiae *[11]. Note that only the high-throughput data contain 'negatives'. The 'strong' CDK consensus is S/T-P-X-R/K, where X represents any amino acid.

**Table 2:** Likelihood ratio tests for spatial clustering of CDK consensus matches

	Number	H_*0 *_(*f*_*s*_, *f*_*w*_)	H_*1 *_(*f*_1*s*_, *f*_1*w*_) (*f*_2*s*_, *f*_2*w*_)	Λ	*P *value
'Known'	12	6.72, 10.8	25.1, 34.3; 2.66, 5.66	44.4	1.2 × 10^-9^
'Unbiased positives'	18	2.81, 8.51	19.8, 31.6; 1.53, 6.77	20.2	1.6 × 10^-4^
'Unbiased negatives'	173	0.67, 6.68	2.93, 47.7; 0.65, 6.34	5.58	0.13
'Known,' scrambled	12	0.96, 6.04	4.60, 10.2; 0.00, 4.48	5.21	0.15

Comparison of a one-component versus two-component mixture of multivariate geometric distributions in different protein sets. Maximum likelihood parameter estimates (in matches per 1,000 residues) under the two hypotheses are indicated by *f*. See text for descriptions of parameters. Λ indicates the likelihood ratio test statistic, which is expected to be χ^2 ^distributed with three degrees of freedom. *P *values are computed under that assumption. Seven low-confidence open reading frames were removed from the 'unbiased negatives', although similar results are obtained if they are included. CDK, cyclin-dependent kinase.

**Table 3:** Correlation between cluster score and position and phosphorylation in the kinase assay

	Correlation (*P *value)		
	
	'Unbiased'	'2+'	'1cc'
*S*_*LR*_	0.54 (4.21 × 10^-14^)	0.34 (1.50 × 10^-11^)	0.03 (NS)
*S*_*BN*_	0.56 (< 2 × 10^-16^)	0.33 (3.03 × 10^-11^)	0.27 (0.0019)
Pos	-0.26 (0.00299)	-0.23 (5.7 × 10^-06^)	-0.02 (NS)
Foldedness	-0.24 (0.00564)	-0.19 (0.000137)	-0.25 (0.00555)
Density	0.43 (2.62 × 10^-10^)	0.18 (0.00049)	0.05 (NS)
*S*_*LR *_+ pos	0.52 (0.00818)	0.37 (0.000552)	(NS)
*S*_*LR *_+ pos + foldedness	0.51 (0.0160)	0.39 (0.00150)	(NS)

We calculated the Pearson correlation between the results of the kinase assay and either likelihood ratio score (*S*_*LR*_), the minimal product of binomial probabilities (*S*_*BN*_), the minimum distance from the either edge of the optimal cluster (identified using *S*_*BN*_) to the closest terminus (pos), the 'foldedness' of the optimal cluster, or simply the density of strong matches per residue (density). To calculate *P *values we used the generalized linear models implemented in R [57]. In addition, we fit linear models to combine the *S*_*LR *_score with the position and foldedness of the cluster (*S*_*LR *_+ pos and *S*_*LR *_+ pos + foldedness). When the variables did not all contribute significantly, we report NS (not significant). For the other sets, the *P *values are for the addition of the least significant term to the model. The total numbers of proteins in each set are slightly smaller than that reported [11] because since the time of that study proteins have been removed from the database and because scores cannot be computed for each gene for each method.
